# TF–RBP–AS Triplet Analysis Reveals the Mechanisms of Aberrant Alternative Splicing Events in Kidney Cancer: Implications for Their Possible Clinical Use as Prognostic and Therapeutic Biomarkers

**DOI:** 10.3390/ijms22168789

**Published:** 2021-08-16

**Authors:** Meng He, Fuyan Hu

**Affiliations:** Department of Statistics, School of Science, Wuhan University of Technology, 122 Luoshi Road, Wuhan 430070, China; hm798104845@whut.edu.cn

**Keywords:** alternative splicing, RNA-binding protein, transcription factor, TF–RBP–AS triplets, regulation mechanism

## Abstract

Aberrant alternative splicing (AS) is increasingly linked to cancer; however, how AS contributes to cancer development still remains largely unknown. AS events (ASEs) are largely regulated by RNA-binding proteins (RBPs) whose ability can be modulated by a variety of genetic and epigenetic mechanisms. In this study, we used a computational framework to investigate the roles of transcription factors (TFs) on regulating RBP-AS interactions. A total of 6519 TF–RBP–AS triplets were identified, including 290 TFs, 175 RBPs, and 16 ASEs from TCGA–KIRC RNA sequencing data. TF function categories were defined according to correlation changes between RBP expression and their targeted ASEs. The results suggested that most TFs affected multiple targets, and six different classes of TF-mediated transcriptional dysregulations were identified. Then, regulatory networks were constructed for TF–RBP–AS triplets. Further pathway-enrichment analysis showed that these TFs and RBPs involved in triplets were enriched in a variety of pathways that were associated with cancer development and progression. Survival analysis showed that some triplets were highly associated with survival rates. These findings demonstrated that the integration of TFs into alternative splicing regulatory networks can help us in understanding the roles of alternative splicing in cancer.

## 1. Introduction

Renal cell carcinoma (RCC) is a common malignant tumor that, according to 2020 global cancer data released by the International Agency for Research on Cancer (IARC), accounts for 2.2% of all new cancer cases, with approximately 431,288 new cases and 179,368 deaths worldwide, and there will be approximately 73,587 new cases and 43,196 deaths in China. There are different types of RCC, and kidney renal clear cell carcinoma (KIRC) is the most common type of RCC, accounting for about 75% of adult RCC malignancies [1]. Since KIRC is radiotherapy- and chemotherapy-resistant, surgery is currently the most effective treatment [2]. Despite early surgical treatment, 30% of patients with a localized tumor eventually develop metastases, and the five-year overall survival rate of metastatic KIRC is only 12% [2,3,4]. Although immune-checkpoint and targeted therapeutics inhibitors have changed the landscape of treatment for KIRC, most patients have never experienced significant clinical benefits [5,6]. Therefore, it is essential to reveal the underlying molecular mechanisms of KIRC and find more powerful diagnostic biomarkers or therapeutic targets.

Alternative splicing (AS) is a pivotal process that increases the diversity of proteins [7]. The destruction of this process may lead to disorders of normal cell functions and eventually develop cancer. The dysregulation of alternative splicing is a new hallmark of cancer and can be used as a biomarker for drug therapy [8,9]. The regulation of alternative splicing is a complicated process, including cis-regulatory elements and trans-acting factors [10,11]. Growing evidence indicates that alternative splicing is strongly associated with kidney cancer. For example, some specific alternative splicing events (ASEs) are potential prognostic biomarkers of kidney cancer [12], some ASEs were predicted to be associated with cancer stemness in KIRC [13], and a novel prognostic index based on prognosis-related AS events was revealed in KIRC [14]. However, comprehensively understanding the alterations of AS in KIRC remains unknown. Thus, exploring the regulatory patterns that control AS provides valuable molecular insights and provides solutions for cancer treatment [15].

ASEs are largely regulated by RNA-binding proteins (RBPs) that can bind to cis-regulatory elements in introns and exons to regulate splicing [16]. Specifically, RBPs recruit different factors and enzymes to form different complexes that bind to specific regulatory sequences of their target pre-mRNA, thus modulating RNA alternative splicing [17,18]. However, the mechanism of interactions between splicing complexes and target pre-mRNA is complex. An RBP can act on hundreds of mRNA target genes, but under the influence of environmental stimuli, an RBP only acts on a subset of its RNA target genes [15], which is often influenced by transcription factors (TFs). By regulating the activity of RBP, TFs can affect downstream alternative splicing outcomes. Therefore, discovering and elucidating the interactions between RBPs, AS events (ASEs), and TFs are necessary for understanding the mechanisms of alternative splicing.

AS could be mediated by nonsense-mediated mRNA decay (NMD), which is an mRNA surveillance pathway in eukaryotic cells that can rapidly degrade mRNAs bearing premature translation termination codon (PTC) to protect cells from adverse effects of truncated proteins [19,20]. Some studies showed that AS coupled to NMD (AS-NMD) as a novel post-transcriptional mechanism is closely related to cancer [21,22]. Thus, studying AS-NMD may provide a novel view on understanding AS mechanisms in kidney cancer.

In this study, we established a computational method for studying the relationships between RBPs, ASEs, and TFs. Our method discovers how alternative splicing outcomes change under the regulation of the same RBP when transcription factor expression is different. A triplet contains three elements: a specific ASE, a specific RBP that may regulate the ASE, and a TF that may change the splicing regulation of the RBP. We first used the TCGA-KIRC dataset to analyze and select key cancer-specific alternative splicing, and differentially expressed RBPs and TFs in cancer; then, we applied linear mixed models to identify triplets in kidney cancer. A regulatory network for triplets in KIRC was established, and the potential mechanisms were explored. Survival analysis showed that these triplets were highly associated with survival rates. The results provided another perspective for further investigations into the molecular pathogenesis of kidney cancer and the selection of the potential therapeutic targets for the treatment of kidney cancer.

## 2. Results

### 2.1. Screening of Key ASEs in Kidney Cancer

**Figure 1:**
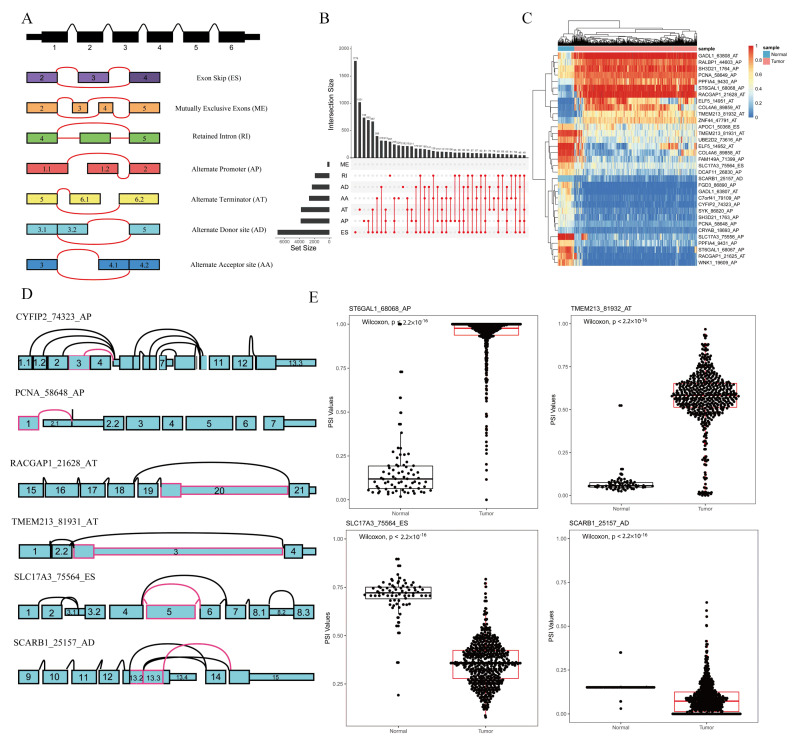
Overview of AS events profiling in KIRC. (**A**) Seven types of AS events: exon skip (ES), mutually exclusive exons (ME), retained intron (RI), alternate promoter (AP), alternate terminator (AT), alternate donor site (AD), and alternate acceptor site (AA). (**B**) UpSet plot of interactions between the seven types of detected AS events (n = 46,415) in KIRC. (**C**) Heat map of significant ASEs (n = 33). Horizontal axis shows clustering information of samples (normal or tumor); left longitudinal axis shows clustering information of ASEs. Gradual change in color from green to red represents PSI value of ASEs altered from low to high. (**D**) Splice graphs of some representative ASEs. Exons were drawn to scale, and connecting arcs represent splice paths. (**E**) Boxplots of four differentially expressed ASEs showing different expressions of AS events between KIRC and normal samples. Wilcoxon test was used for data comparison.

The file of ASEs was composed of PSI values of 46,415 ASEs involved 10,600 genes for 533 KIRC patients and 77 normal samples. According to their splicing pattern, these ASEs were divided into seven types: exon skip (ES), mutually exclusive exons (ME), retained intron (RI), alternate promoter (AP), alternate terminator (AT), alternate donor site (AD), and alternate acceptor site (AA), which are illustrated in Figure 1A. Furthermore, an UpSet plot was generated to visualize the intersecting sets of each AS type, as shown in Figure 1B.

By comparing the expression of ASE in KIRC and normal samples, 33 ASEs were identified as differentially expressed ASE through the iterative MI-SIS method, among which there were 19 APs, 2 ESs, 11 ATs, and 1 AD. Detailed information on the differentially expressed ASEs is listed in Table 1. A receiver-operating-characteristic (ROC) curve was drawn to show the prediction accuracy of our method, as shown in Supplementary Figure S1, which suggested that our method reached a high area under the curve (AUC = 0.993). A heat map was used to elaborate the expression differences of the 33 differentially expressed ASEs between the tumor and normal samples, as shown in Figure 1C. Some of the differentially expressed ASEs are depicted as splice graphs, which summarize the transcript variations into directed acyclic graphs, and represent exons as rectangular nodes and splice junctions as edges (Figure 1D). Furthermore, the boxplots of four differentially expressed ASEs are shown in Figure 1E. Both the heat map and boxplots suggested that the 33 differentially expressed ASEs could be used as diagnostic biomarkers for KIRC.

### 2.2. Identification of Differentially Expressed RBPs and TFs in Kidney Cancer

The method of log2FC and the Wilcoxon test were used to identify differentially expressed TFs and RBPs from 1635 RBPs and 1826 TFs. Lastly, 302 TFs and 177 RBPs were selected under the criteria of *p* value < 0.05 and |log2FC| > 1. All are shown in Supplementary Tables S1 and S2.

### 2.3. Category of TF Action

Depending on whether the correlation of RBP-AS increased or decreased in two “abundance” groups (low to high) of TFs, three possible modes of TFs action were identified: “attenuates interaction”, “enhances interaction”, and “inverts interaction”. Among them, each mode comprised two subtypes: strengthen attenuation interaction (SAI), weaken attenuation interaction (WAI); strengthen enhancement interaction (SEI), weaken enhancement interaction (WEI); and invert positive to negative (IPN), invert negative to positive (INP). These cases and details interpretations are listed in Table 2.

### 2.4. Detection of TF–RBP–AS Triplets in Kidney Cancer

**Figure 2:**
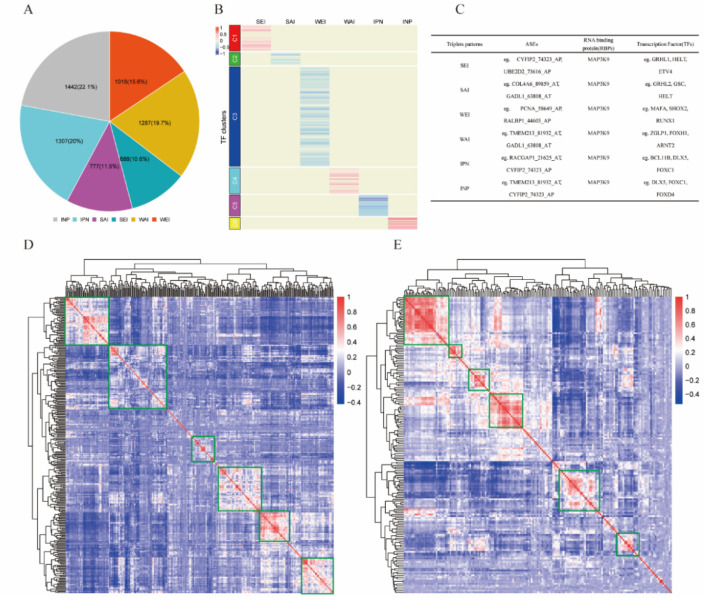
Identified triplets in KIRC. (**A**) Six mode subcategories of TFs according to correlation between expression of RBP and PSI value of ASE. Number in the pie chart means the percentage of each subcategory. (**B**) Clusters by the regulation patterns of TFs that are involved in the triplets containing the RBP MAP3K9. Six clusters were grouped according to TF subcategories. (**C**) Information of some representative triplets involved MAP3K9. (**D**) Correlation heat map of TFs involved in 6519 triplets. (**E**) Correlation heat map of RBPs involved in 6519 triplets.

Linear mixed-effects models were employed to identify TF–RBP–AS triplets for differentially expressed TFs (n = 302), RBPs (n = 177), and ASEs (n = 33) based on Formula (1); 6519 TF-mediated significant triplets were statistically significant under our criteria as described in Section 4 (Supplementary Table S3). The 6519 TF–RBP–AS triplets included 290 TFs, 175 RBPs, and 16 ASEs corresponding to 13 genes. According to the correlation changes between RBP and ASE expressions under different abundance groups (low or high) of TF, six subcategories were identified: 688 triplets in SEI, 1018 triplets in WEI, 777 triplets in SAI, 1287 triplets in WAI, 1307 triplets in IPN, and 1442 triplets in INP (Figure 2A).

### 2.5. Construction of Splicing-Regulatory Network for Triplets

**Figure 3:**
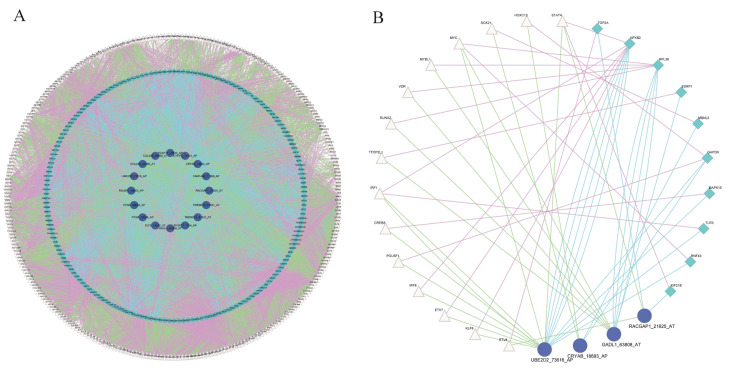
Construction of protein–protein interaction network of triplets. (**A**) Protein–protein interaction network of 6519 triplets. Pink triangles represent TFs, green rhombuses represent RBPs, purple circles represent ASEs. Purple lines represent relationships between TF and RBP genes, green lines represent relationships between TF genes and ASEs, and blue lines represent the relationships between RBP genes and ASEs. (**B**) Triplet network involved in known PPI network in STRING.

We constructed a splicing-regulatory network for 6519 triplets. The splicing-regulatory network was visualized in Cytoscape. The relationships between TF and RBP (purple lines), TF and ASE (green lines), and RBP and ASE (blue lines) are exhibited (Figure 3A). The complexity of alternative splicing regulation can be seen from the triplet network. In addition, we compared this splicing-regulatory network with the known human PPI network in STRING and found that 18 triplets were connected in it as shown in Figure 3B.

In the network of 18 triplets, IRF1, NFKB2, and UBE2D2_73616_AP were the TF, RBP, and ASE with the highest node degree, respectively. Interferon regulatory factor 1 (IRF1) is a tumor-suppressor gene that is associated with RCC, which can promote the apoptosis of tumor cells and increase tumor-cell sensitivity to chemotherapeutic drugs [23]. Nuclear factor kappa B subunit 2 (NFKB2) regulates all important aspects of RCC biology, including resistance to apoptosis, angiogenesis, and multidrug resistance [24]. Ubiquitin conjugating enzyme E2 D2 (UBE2D2) plays an important role in the development of breast cancer [25]. These results showed that the triplet network may play key roles in the development of RCC.

### 2.6. Survival and Functional-Enrichment Analyses for Triplets

**Figure 4:**
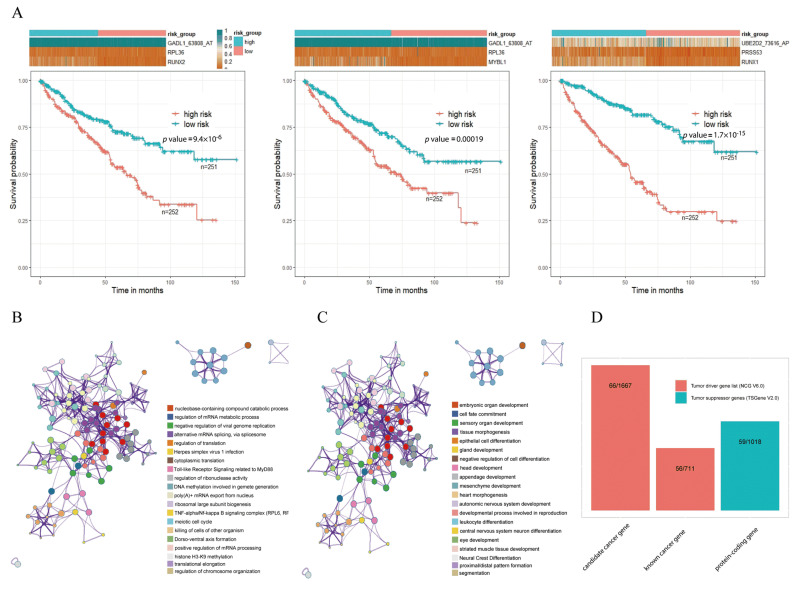
Functional analysis of TF–RBP–AS triplets. (**A**) Survival analysis of three representative triplets. Red line represents the high-risk subgroup, and green line represents the low-risk subgroup. (**B**) Enrichment analysis of TF genes involved in 6519 triplets. (**C**) Enrichment analysis of RBP genes involved in 6519 triplets. (**D**) Cancer-relevant modulators identified according to the Network of Cancer Genes (NCG) and Tumor Suppressor Gene (TSGene) databases (* using Metascape for gene-enrichment analysis).

To investigate the association between triplets and kidney cancer, we performed survival analysis in tumor samples by constructing prognostic-risk-score models through Formula (2). For each triplet, on the basis of the expression of its components (i.e., an RBP, a TF, and an ASE), each tumor sample had a calculated risk score. Then, all tumor samples were divided into high- and low-risk subgroups with the median value of risk scores as cutoff. Then, survival analysis was carried out for the 6519 triplets, and the results suggested that there were significant survival differences between the high- and low-risk subgroups for 5580 triplets (Supplementary Table S4). For example, although GADL1_63808_AT and UBE2D2_73616_AP were two of the ASEs that did not show too much differential expression, survival analysis indicated that triplets including GADL1_63808_AT and UBE2D2_73616_AP in KIRC were significantly associated with overall survival outcomes by comparing the high- and low-risk subgroups (Figure 4A). Survival plots of three representative triplets, namely RUNX2-RPL36-GADL1_63808_AT, MYBL1-RL36-GADL1_63808_AT, and RUNX1-PRSS53-UBE2D2_73616_AP, are shown in Figure 4A, suggesting that the high-risk subgroup had a worse survival rate for each triplet (log-rank *p* values were 9.4 × 10^−6^, 0.00019 and respectively 1.7 × 10^−15^). The results of survival analysis suggested that the detective triplets could serve as prognostic biomarkers for kidney cancer.

Pathway-enrichment analysis showed that these TFs and RBPs were highly enriched in categories that are associated with cancer development and progression, including herpes simplex virus 1 infection, meiotic cell cycle, and the negative regulation of cell differentiation (Figure 4B,C). In addition, cancer-relevant modulators were identified through a tumor-associated gene list from the Network of Cancer Genes (NCG, v6.0) [26] and the Tumor Suppressor Gene (TSGene, v2.0) databases [27], separately (Figure 4D). The 2378 tumor diver genes obtained from NCG were 711 known cancer genes and 1667 candidate cancer genes. Among all genes involved in the 6519 triplets, 122 genes overlapped with tumor diver genes, almost reaching 25.58% (122/477) of the total numbers of triplet genes. Approximately 12.37% (59/477) of the genes were tumor-suppressor genes. These results suggested that these triplets were involved in the occurrence and development of cancer.

### 2.7. Analysis of Splicing Event of PCNA_58648_AP Influenced by Triplets in Kidney Cancer

**Figure 5:**
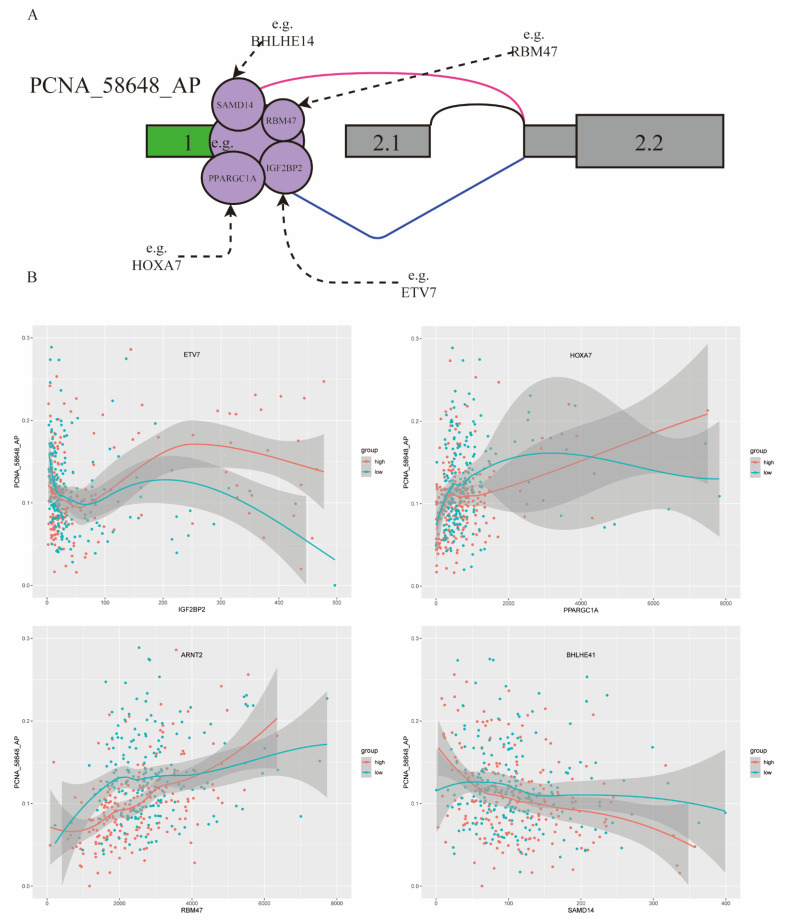
PCNA_58648_AP influenced by TFs and RBPs involved in triplets in KIRC. (**A**) Examples of some TFs and RBPs involved in triplets. (**B**) Four triplets influencing splicing of PCNA_58648_AP: four TFs (ETV7, HOXA7, ARNT2, BHLHE41) and four RBPs (IGF2BP2, PPARGC1A, RBM47, SAMD14). Red, samples in high TF expression group (top 40%); green, samples in low TF expression group (bottom 40%). X axis is the expression level of RBP, and Y axis is the PSI value of PCNA_58648_AP.

In this study, 80 triplets were found to be involved in the ASE of PCNA_58648_AP, including 28 TFs and 58 RBPs. Previously studies reported that PCNA had obvious differential expression in RCC and played an important role in cell proliferation [28]. Some triplets of PCNA_58648_AP are shown in Figure 5A.

For example, ETV7 is a modulator of IGF2BP2 and may change the role of IGF2BP2 on PCNA_58648_AP. In the low ETV7 expression group, correlation between IGF2BP2 expression and PCNA_58648_AP splicing level was −0.28, while such correlation became 0.14 when in the high ETV7 expression group. HOXA7, as a modulator of PPARGC1A, enhanced the splicing regulation on PCNA_58648_AP. In the low HOXA7 expression group, correlation between PPAGGC1A expression and PCNA_58648_AP splicing level was 0.23, while such correlation became 0.51 in the high HOXA7 expression group. In addition, ARNT2 had a similar effect on RBM47, and BHLHE41 had a similar effect on SAMD14 (Figure 5B). Thus, the results showed that differentially expressed TFs changed the role of RBPs on regulating PCNA_58648_AP, and these triplets could be diagnostic and prognostic biomarkers of KIRC. This way of regulation can provide some insights into the dysregulation of splicing results in many diseases, not only KIRC.

## 3. Discussion

In this study, we proposed to use a linear mixed-effects model to identify TF–RBP–AS triplets with which the expression level of TFs was associated in changing the targets AS outcomes of RBPs in KIRC. A computational method was previously developed to identify modulators whose expression levels could affect the relationship between the RBPs and its target alternative splicing outcomes, and this only focused on target splicing outcomes of QKI that can be influenced by the expression level of modulators [15]. In addition, several computational methods were developed for identification modulators whose expression levels could affect the regulation activity of TFs toward its target genes [29,30], and these studies reported that the expression level of modulators can affect the transcriptional activities of TFs. The unique ability of our method is that it can identify triplets by considering the influence of some objective factors and discovering how the expression level of TFs is associated with changing the target AS outcomes of RBPs. Our method aimed to discover the impact of TFs on AS after acting on RBPs and provides a new perspective for studying the network of alternative splicing mechanisms in cancers.

Not all ASEs are necessarily related to cancer, so it is an effective way to find a subset of cancer-specific ASEs by comparing the PSI values of the ASE between normal and tumor samples. Then, 33 ASEs corresponding to 23 genes were found by using the method of iterative MI-SIS. A literature review found 10 (TMEM213, ELF5, PCNA, RALBP1, WNK1, SLC17A3, APOC1, SCARB1, DCAF11, CRYAB) out of 23 genes to be related to kidney cancer [28,31,32,33,34,35,36,37,38,39]. In particular, ELF5, SLC17A3, RALBP1, WNK1, APOC1, and CRYAB were experimentally verified to play an important role in the occurrence and development of kidney cancer. ELF5 is a tumor-suppressor gene for RCC; SLC17A3 is related to the origin of RCC; RALBP1 plays an oncogenic role in RCC; WNK1 promotes renal tumor progression by activating TRPC6-NFAT pathway; APOC1 is significantly correlated with RCC tumor size and histological grade, and CRYAB promotes RCC tumor angiogenesis by increasing vascular survival during tube morphogenesis. On the basis of the role of these genes in kidney cancer, the influence of gene expression on kidney cancer is important. Some of the remaining 13 genes also play important roles in kidney cancer. For example, COL4A6 is related to hereditary nephropathy, and RACGAP1 can promote the proliferation and suppress apoptosis of renal tubular cells [40,41]. This evidence indicated that these ASEs are closely related to RCC.

In our study, 302 differentially expressed TFs, 177 differentially expressed RBPs, and 33 cancer-specific ASEs were formed to the combinations of TF–RBP–AS; all of them were screened by linear mixed-effects models to obtain significant TF–RBP–AS triplets for further study. Lastly, 6519 TF–RBP–AS triplets were identified as significant TF–RBP–AS triplets. These triplets included 290 TFs, 175 RBPs, and 16 ASEs corresponding to 13 genes. The results showed that the functions of TFs are very complicated, and even the same TF may play different or opposite roles on the same RBP targets. For example, LYL1 plays an enhanced role on the C2orf15-TMEM213_81931_AT pair, but LYL1 inverts the splicing activity of C2orf15 on RACGAP1_21625_AT. Detailed information can be found in Supplementary Table S2. Results demonstrated the complexity of the alternative splicing regulation mechanism. Among the 16 ASEs involved in 6519 triplets, PCNA_58648_AP received more attention because previous studies showed that PCNA has significant association with RCC. Eighty TF-RBP-PCNA_58648_AP triplets were identified, including 28 TFs and 58 RBPs, and LYL1-RELB-PCNA_58648_AP and HOXA7- PPARGC1A-PCNA_58648_AP were two of them. LYL1 gene amplification was associated with the upregulation of cancer-related pathways in uterine corpus endometrial cancer, and RELB gene plays an oncogenic role in colorectal cancer [42,43]. Our results showed that the high expression of LYL1 tended to attenuate RELB regulation role on PCNA_58648_AP. Correlation between RELB expression and PCNA_58648_AP PSI was −0.18 in the low LYL1 expression group, and such correlation changed into −0.06 in the high LYL1 expression group. HOXA7-PPARGC1A- PCNA_58648_AP was another inferred triplet. HOXA7 is associated with the metastasis of liver cancer [44]. PPARGC1A overexpression promotes lung-cancer metastasis [45]. When HOXA7 expression was low, correlation between PPARGC1A expression and the PSI of PCNA_58648_AP was 0.51, while such correlation turned into 0.23 when HOXA7 expression became high.

Two ASEs (i.e., COL4A6_89859_AT and GADL1_63808_AT) in the triplets were the fragments of the targeted RNA of NMD, which is a mechanism to degrade mRNA transcripts containing PTC [19]. COL4A6 and GADL1 are related to cancer. The downregulation of COL4A6 was associated with prostate cancer progression and metastasis, and the overexpression of GADL1 was associated with cancer cell migration and morphology (including cell area, thickness, volume, perimeter length, irregularity, and eccentricity) [46,47]. The expression of COL4A6 and GADL1 may be regulated by alternative splicing-coupled NMD to promote the development of cancer. This evidence indicated that alternative splicing-coupled NMD is related to RCC.

On the basis of the 6519 inferred triplets, we constructed an interaction network using TF–RBP–AS triplets and further refined the network by only including 18 TF–RBP–AS triplets that had documented interactions from the STRING database. Some of the interactions had already been reported by other studies. For example, IRF1 could inhibit NFKB2 activity to induce breast cancer cell-specific growth inhibition [48]. MYC can also suppress NFKB2 to accelerate lymphomagenesis, and IRF1 can bind to TLR3 to regulate transcriptional activation in cellular antiviral activities [49,50]. Then, the study of the IRF1-NFKB2-UBE2D2 _73616_AP triplets may have discovered their regulatory mechanism in RCC. IRF1 is a tumor-suppressor gene that is associated with RCC [51,52]. NFKB2 is involved in inflammation and immune function and participates in activating the Toll-like receptor 4 (TLR4) signaling pathway [53,54]. The UBE2D2 gene regulates the degradation of misfolded, damaged, or short-lived proteins, which occurs via the ubiquitin (Ub)-proteasome system (UPS), and can inhibit TLR4 signaling [55,56]. The NFKB2 and UBE2D2 genes are related to TLR4. The TLR4 gene plays a fundamental role in pathogen recognition and the activation of innate immunity, is required for IRF1 expression, and the TLR4-IRF1 pathway plays important roles in many diseases [57,58]. UBE2D2 inhibits the expression of IRF1, while NFKB2 promotes the expression of IRF1. When the expression of IRF1 changes from low to high, UBE2D2 and NFKB2 have an enhanced negative correlation trend, which is what we inferred. UBE2D2 and NFKB2 may go through TLR4 to influence IRF1 to destroy the immune system and promote the occurrence and development of cancer. This evidence indicated that interactions in the identified triplets play key roles in the development of RCC and could be drug targets for in RCC.

We performed additional dry lab experiments to prove that the triplets we identified could reveal the mechanism of alternative splicing. We compared interactions in our 6519 identified triplets with protein–protein interactions (PPIs) obtained from the STRING database. The results showed that the 4456 TF-RBP pairs, 1509 TF-ASE pairs, and 1280 RBP-ASE pairs involved in the triplet had 275 TF-RBP pairs, and 55 TF-ASE pairs and 93 RBP-ASE pairs overlapped with PPI, respectively, which is shown in Supplementary Figure S2. We also further confirmed interactions in the triplets through the following two aspects. Firstly, we compared the interactions of TF-ASE/RBP in the triplets with known interactions of TF targets. We combined the research results of Zhang [59] and Han [60] on transcription factors and transcription factor target genes and obtained a dataset of 970 transcription factor target genes. This dataset contained 167 transcription factors involved in our triplets. We found that 3815 triplets involved the 167 transcription factors from 6519 triplets and compared them with the 970 transcription factor target genes. The results suggested that RBPs in 163 out of 2593 TF-RBP pairs were confirmed by the 970 transcription factor target genes, and genes corresponding to ASEs in 159 out 861 TF-ASE pairs were confirmed by the 970 transcription factor target genes, which are shown in Supplementary Figure S3. Secondly, in order to verify RBP-ASE pairs, we used the results of Paz’s [61] study on the binding site of RBP and RNA. However, only eight RBPs involved in 6519 triplets had information of the binding site from Paz’s study, so we only compared 293 triplets involving 64 RBP-ASE pairs with Paz’s results. Supplementary Tables S5 and S6 show that 24 of 64 RBP-ASE pairs found that RBP had binding sites on ASE. Of the 293 triplets, 145 were in involved in these 24 RBP-ASE pairs, suggesting that the triplets we inferred could reveal the underlying mechanisms of alternative splicing.

In order to investigate whether TF–RBP–AS triplet signatures could act as independent prognostic markers, survival analysis was also performed on each of the triplets on the basis of the results of our constructed prognostic risk score models. Survival analysis results showed that 85.60% (5580/6519) of the triplets were significantly related to the overall survival (OS) of RCC patients. Additionally, although the combination of TF and RBP expression and the PSI value of ASE successfully stratified patients, approximately 83.76% (4674/5580) of the triplets included the component that was not significantly associated with KIRC prognosis. This evidence indicated that these triplets could act as risk predictors of RCC, and network-based biomarkers are expected to be more effective and provide deep insights into the molecular mechanism of RCC progression.

Pathway-enrichment results showed that all TF and RBP genes involved in 6519 triplets were enriched in cancer-development- and progression-related pathways, including herpes simplex virus 1 infection, meiotic cell cycle, and the negative regulation of cell differentiation. Herpes simplex virus 1 (HSV-1) infection is a risk factor in the development of human malignancies which can induce apoptosis in neighboring human cancer cells and can be used as a potential target to improve anticancer activity [62,63]. Meiotic errors of the cell-cycle process are an important characteristic of kidney cancer [64,65], and cell differentiation is involved in the process of many human cancers such as RCC [66,67]. This evidence indicated that these TFs and RBPs are involved in the occurrence and development of RCC and could serve as targets for the treatment of RCC.

Although a model was established in this study, and TFs were integrated into regulatory networks to help improve the understanding of the regulatory network of AS, there are some limitations. Firstly, the roles of triplets acing as a whole need to be further be further confirmed by experiments. Moreover, the function and mechanism of how TF changes RBP regulation on AS need to be further studied by experiments. Lastly, our study can provide a perspective for understanding the regulatory mechanism of alternative splicing in cancer, and the corresponding results are helpful for the treatment for RCC.

## 4. Materials and Methods

### 4.1. Data Acquisition and Processing

Data on mRNA splicing patterns of KIRC were downloaded from the TCGA SpliceSeq portal (https://bioinformatics.mdanderson.org/TCGASpliceSeq) (accessed on 9 February 2020). TCGA SpliceSeq is a web-based bioinformatics resource with information of mRNA splicing patterns of 33 different tumors, which provide the PSI value for each splicing event in each cancer sample [68]. The PSI value uses a ratio to quantify the expression of splicing events [69]. The name of each ASE is composed of the gene symbol, ID number, and splicing type, for example, RACGAP1_21625_AT. In total, 72 normal samples and 533 KIRC samples were enrolled in the analysis of ASEs, and the mean value of each AS in the normal and KIRC samples was used to replace the missing values of each ASE. We also downloaded full clinical follow-up information data of 537 patients through TCGA Data Commons (https://gdc.cancer.gov/) (accessed on 8 October 2020) for the KIRC cohort.

The expression datasets, including 20,501 genes for 72 normal samples and 529 KIRC samples, were downloaded from The Cancer Genome Atlas (TCGA, http://cancergenome.nih.gov/) (accessed on 24 July 2020). After removing genes that did not express in more than 50% samples, 18,103 genes were left for further study. The list of 1826 RBPs was obtained from hRBPome (http://caps.ncbs.res.in/hrbpome/) (accessed on 28 June 2020), and each RBP had to be reported by at least two studies. The list of 1635 TFs came from a review of human transcription factors [70].

We downloaded protein–protein interaction (PPI) datasets from the STRING database version 10.5 (http://string-db.org) (accessed on 23 November 2020), which included direct (physical) and indirect (functional) interactions.

### 4.2. Identify Cancer-Specific Alternative Splicing Events

The mutual information-sure independence screening (MI-SIS) method [71] was used to identify cancer-specific ASEs in our curated datasets. The mutual information value between ASE and the classification of samples (i.e., tumor or normal samples) was first computed for each ASE. Then, 50 ASEs with the highest mutual information value were identified. On the basis of the top 50 ASEs, an event set composed of these 50 events was processed to the next screening process by using the iterative sure independence screening (ISIS) with tenfold cross-validation (CV) method, and LASSO as the penalty function for intermediate penalized likelihood estimation to form the final set of those highly related to cancer ASEs. Details of the MI-SIS method can be found in Supplementary Materials.

### 4.3. Identification of Differentially Expressed RBPs and TFs

The expression data of 1826 RBP genes and 1635 TF genes were screened from the mRNA expression profiles. Then, log2FC and Wilcoxon test were used to select differentially expressed RBPs and TF genes between tumor and normal samples. Only genes with an absolute value of log2FC larger than 1 (|log2FC| > 1) and adjusted *p* value < 0.05 (Wilcoxon test) were selected as differentially expressed RBPs and TFs.

### 4.4. Construction of TF–RBP–AS Triplets in KIRC

In this study, a linear mixed-effects model as shown in (1) was used to build TF–RBP–AS triplets. The model was as follows:

(1) $$\left\{ \begin{array}{c} Y_{AS}=\beta_{0}+\beta_{1}X_{RBP}+\beta_{2}X_{TF}+\beta_{3}X_{RBP}*X_{TF}+\beta_{4}X_{gender}+\beta_{5}X_{stage}+b_{1}Z_{age}+b_{2}Z_{race}+b_{3}Z_{year}+\varepsilon ,\\ b_{i}\sim N(0,D),i=1,2,3,\varepsilon \sim N(0,\Sigma ) \end{array}\right.$$

where $Y_{AS}$ is the PSI value of an ASE, which is the dependent variable. $X_{RBP}$ and $X_{TF}$ are the expressions of an RBP and a TF, respectively, and $X_{gender}$ and $X_{stage}$ are the age at initial pathologic diagnosis and the year of initial pathologic diagnosis, respectively. $X_{RBP}$, $X_{TF}$, $X_{gender}$ and $X_{stage}$ are the fixed effects. $Z_{age}$, $Z_{race}$, and $Z_{year}$ are age at diagnosis, race, and year of diagnosis, respectively, which are random effects. $\beta_{3}$ represents the interactive effect of RBP and TF. If the interaction of RBP and TF affects AS, then $\beta_{3}$ is expected to be nonzero.

**Figure 6:**
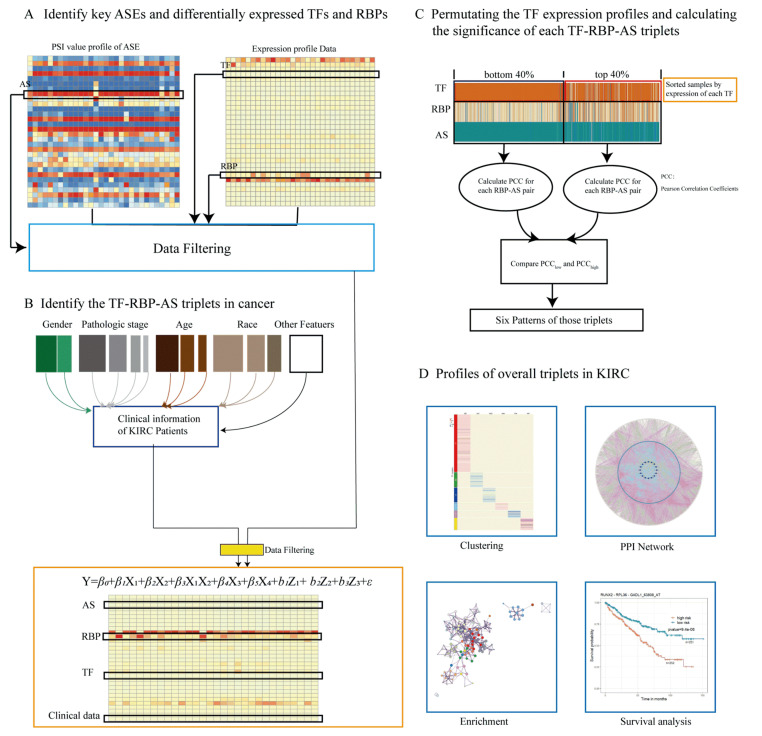
Research methodology. (**A**) Key ASEs and differentially expressed RBPs and TFs were identified, expression data of RBP and TF and PSI value of ASE were integrated, relevant clinical information of the patient from the data of TCGA-KIRC was extracted, and all data were integrated. (**B**) The linear mixed-effects model was used to predict triplets. Only triplets with significant *β*_1_, *β*_2_, and *β*_3_ *p* values were considered and selected for the following analysis. Each triplet contains three objects: the expressions of TF and RBP and the PSI value of ASE. (**C**) For each triplet, we grouped samples into “low” and “high” groups on the basis of the expression level of TF (bottom/top 40% samples) in the specific triplet, and we compared Pearson’s correlation coefficient values of RBP expression and PSI value of ASE in two groups, identifying TF function categories. (**D**) In order to explore the prognostic value of these triplets, clustering analysis was performed, and a PPI network was constructed; enrichment and survival analyses were carried out.

Only when the *p* values of RBP, TF, and the interaction term of RBP and TF were less than 0.05, and the coefficient of the interaction term of RBP and TF was nonzero, were such TF–RBP–AS triplets considered to be statistically significant. Details of this study design are illustrated in Figure 6.

### 4.5. Protein–Protein Interaction Network Analysis

The protein–protein interaction network in which all TF–RBP–AS triplets were involved was constructed and plotted by Cytoscape (https://cytoscape.org/) (accessed on 20 November 2020). In addition, the triplet network that was involved in the known PPI network in STRING was plotted by Cytoscape.

### 4.6. GO Functional and KEGG Pathway Enrichment Analyses of Genes

TF and RBP genes contained in TF–RBP–AS triplets were selected to perform gene ontology (GO) and Kyoto Encyclopedia of Genes and Genomes (KEGG) pathway-enrichment analysis using Metascape (https://metascape.org/gp/index.html#/main/step) (accessed on 4 January 2021). A *p* value less than 0.05 was statistically significant.

### 4.7. Establishment of Triplet Signature for KIRC Prognosis

We used the R survival package and coxph function for survival analysis. RBPs, TFs, and ASEs in TF–RBP–AS triplets were integrated into triplet signatures through calculating risk scores with the following formula:

(2) $$\mathrm{RS}=\mathrm{Coefficient}\;\mathrm{of}\;\mathrm{ASE}*\mathrm{PSI}\;\mathrm{value}\;\mathrm{of}\;\mathrm{ASE}+\sum_{i=1}^{2}\mathrm{Coefficient}\;\mathrm{of}\;Gene(i)*\;\mathrm{Expression}\;\mathrm{of}\;Gene(i).$$

where *RS* is a triplet signature risk score, short for “risk score”; *Coefficient of ASE* is the regression coefficient of *ASE Gene* in the model of univariate Cox regression; *PSI value of ASE* is the *PSI value* of *ASE* for the sample. *Coefficient of Gene* is the regression coefficient of the TF or RBP gene in the model of univariate Cox regression; *Expression of Gene* is the expression value of the TF or RBP gene. On the basis of this formula, each sample could obtain a triplet signature risk score. Then, the median risk score was used as the cutoff to divide patients into high- or low-risk subgroups. A Kaplan–Meier (KM) survival curve was created and the log-rank test was then performed to compare survival between the high- and low-risk groups.

### 4.8. Statistical Analysis and Software

Data were analyzed and visualized using R statistics software version 3.6.1 and ggplot2 package. Correlations were assessed using Pearson’s correlation coefficient.

## 5. Conclusions

Our computational method identified TFs whose expression levels could affect the relationship between RBPs and target alternative splicing outcomes. Using this method, we identified 6519 significant TF–RBP–AS triplets, including 290 TFs, 175 RBPs, and 16 ASEs. Regulatory networks constructed for TF–RBP–AS triplets explained the mechanism of the dysregulation of AS by TF-dependent RBP. Pathway enrichment analysis indicated that these triplets were highly correlated with the development of RCC. Therefore, they could be used as therapeutic targets in novel treatment strategies in kidney cancer. Finally, the survival analysis based on risk score models in our study seems to indicate that triplets could also serve in the future as prognostic markers in RCC cases. Future research is needed to confirm this hypothesis and promising data.

## Figures and Tables

**Table 1:** Detailed information of 33 cancer-specific ASEs.

Symbol	Gene	AS Type	Exons	From Exon	To Exon	Mean_T	Mean_N	Mi Value
Upregulated								
PPFIA4_9430_AP	PPFIA4	AP	19.1			0.816	0.345	0.354
ST6GAL1_68068_AP	ST6GAL1	AP	1			0.932	0.161	0.347
RACGAP1_21628_AT	RACGAP1	AT	20			0.944	0.216	0.341
TMEM213_81932_AT	TMEM213	AT	4			0.565	0.070	0.340
SH3D21_1764_AP	SH3D21	AP	4.1			0.924	0.600	0.338
GADL1_63808_AT	GADL1	AT	12.2			0.986	0.549	0.338
ELF5_14951_AT	ELF5	AT	4.2			0.648	0.026	0.336
PCNA_58649_AP	PC	AP	2.1			0.884	0.631	0.333
ZNF44_47791_AT	ZNF44	AT	6.2			0.652	0.177	0.328
RALBP1_44603_AP	RALBP1	AP	2			0.878	0.455	0.325
COL4A6_89859_AT	COL4A6	AT	52			0.708	0.054	0.322
Downregulated								
PPFIA4_9431_AP	PPFIA4	AP	1			0.180	0.655	0.354
ST6GAL1_68067_AP	ST6GAL1	AP	3			0.068	0.839	0.347
C7orf41_79109_AP	C7orf41	AP	2			0.047	0.470	0.346
FGD3_86890_AP	FGD3	AP	3			0.019	0.589	0.342
RACGAP1_21625_AT	RACGAP1	AT	21			0.056	0.784	0.341
TMEM213_81931_AT	TMEM213	AT	3			0.435	0.931	0.340
WNK1_19609_AP	WNK1	AP	5			0.036	0.736	0.340
SH3D21_1763_AP	SH3D21	AP	1			0.076	0.408	0.339
GADL1_63807_AT	GADL1	AT	15			0.014	0.451	0.338
ELF5_14952_AT	ELF5	AT	8			0.352	0.974	0.336
PCNA_58648_AP	PC	AP	1			0.116	0.369	0.333
SLC17A3_75564_ES	SLC17A3	ES	5	4	6	0.358	0.707	0.331
APOC1_50368_ES	APOC1	ES	4:5.1	3.2	7	0.424	0.692	0.331
SCARB1_25157_AD	SCARB1	AD	13.3	13.2	15	0.089	0.151	0.326
SYK_86820_AP	SYK	AP	2			0.079	0.456	0.324
SLC17A3_75556_AP	SLC17A3	AP	2			0.199	0.970	0.323
UBE2D2_73616_AP	UBE2D2	AP	2			0.395	0.780	0.322
COL4A6_89858_AT	COL4A6	AT	51.2			0.292	0.946	0.322
DCAF11_26830_AP	DCAF11	AP	2.1			0.262	0.621	0.321
CYFIP2_74323_AP	CYFIP2	AP	3			0.030	0.371	0.319
CRYAB_18693_AP	CRYAB	AP	5.1			0.060	0.224	0.318
FAM149A_71399_AP	FAM149A	AP	3			0.288	0.867	0.318

**Table 2:** Categories of TF-mediated RBP regulations on target AS.

Pattern	PCC_low_	PCC_high_	ΔPCC	Subtype Mode
Enhances	–	––	|PCC_low_| < |PCC_high_|	Strengthens attenuation interaction (SAI)
Attenuates	––	–	|PCC_low_| > |PCC_high_|	Weakens attenuation interaction (WAI)
Inverts	+	–		Inverts positive to negative (IPN)
Inverts	–	+		Inverts negative to positive (INP)
Enhances	+	++	|PCC_low_| < |PCC_high_|	Strengthens enhancement interaction (SEI)
Attenuates	++	+	|PCC_low_| > |PCC_high_|	Weakens enhancement interaction (WEI)

“+” and “–” signs in the columns indicate positive and negative values of Pearson correlation coefficient, and “++” and ‘‘––’’ indicate a larger absolute value of Pearson correlation coefficient compared to its control group.

## Data Availability

Not applicable.

## Supplementary Materials

The following are available online at https://www.mdpi.com/article/10.3390/ijms22168789/s1, Figure S1: receiver operating characteristic curve of 33 ASEs; Figure S2: gene pair relationship comparison of PPI and triplets; Figure S3: alignment results of transcription factor target genes; Table S1: details of differentially expressed RBPs in kidney cancer; Table S2: details of differentially expressed TFs in kidney cancer; Table S3: details of 6519 TF-mediated significant triplets; Table S4: detailed information on 5580 triplets with significant survival differences; Table S5: comparison results of eight RBPs with binding sites; Table S6: 145 triplets which RBP has binding sites on ASE. Supplementary Materials: details of iterative MI-SIS method.
