# Supplementary material for: TF–RBP–AS Triplet Analysis Reveals the Mechanisms of Aberrant Alternative Splicing Events in Kidney Cancer: Implications for Their Possible Clinical Use as Prognostic and Therapeutic Biomarkers
Source: Int J Mol Sci. 2021 Aug 16;22(16):8789. doi: 10.3390/ijms22168789 (PMC8395830; doi:10.3390/ijms22168789)
Supplement: Supplementary file 1 [file ijms-22-08789-s001.zip › Supplementary Methodsú║Details of iterative MI-SIS method.pdf]

Supplementary Methods: Details of Iterative MI-SIS Method

The method of iterative Mutual information (MI)-Sure Independence Screening (SIS) was used to select pivotal ASEs for KIRC. The details of iterative MI-SIS method are as follows:

1.1. The Concept And Estimation of Mutual Information

The Mutual information (MI) of the random variable X and Y is defined by:

$$\mathrm{MI}(\mathrm{X},\mathrm{Y}) = \int p(x,y)log(\frac{p(x,y)}{p(x)p(y)})\,dx\,dy \qquad (1)$$

Here, $p(X)$, $p(Y)$ is probability marginal density function and $p(X,Y)$ is the joint density function. In our study, X represents the AS events, and Y represents the classification of the sample.

The probability density function is not known in practice, and the best we can do is to use the empirical distribution function to estimate it.

For each AS events, PSI values were sorted in ascending order to get the ordered X(1) …X(n) samples. Then, we can get the definition:

$$f(\mathrm{x}) = \begin{cases} 0, & x < x_1 \\ \frac{k}{n}, & x_k \le x < x_{(k+1)}\,, k = 1, \dots n-1 \\ 1, & x \ge x_n \end{cases} \qquad (2)$$

According to the above definition, we can get the distribution law of X as follows:

X	x_1	x_2	$\cdots$	x_n
$\mathrm{P}(X)$	$\frac{1}{n}$	$\frac{1}{n}$	$\cdots$	$\frac{1}{n}$

(3)

The random variable Y which represents the sample type is a two-point distribution. Therefore, its law of distribution is as follows:

Y	0	1
$P(Y)$	$\frac{n-m}{n}$	$\frac{m}{n}$

(4)

Here, 0 represents the normal samples; 1 represents tumor samples, and m represents the number of samples of kidney cancer patients and n represents the total number of samples.

The estimated value of mutual information can be calculated by the following formula:

$$\widehat{MI}(X,Y) = \sum_{k_1=x_1}^{x_n}\sum_{k_2=0}^{1}\hat{p}(X=k_1,Y=k_2)log(\frac{\hat{p}(X=k_1,Y=k_2)}{\hat{p}(X=k_1)\hat{p}(Y=k_2)}) \qquad (5)$$

Here, $\hat{p}(X=k_1,Y=k_2) = \frac{1}{n}\sum_{i=1}^{n} I(x_i=k_1)I(y_i=k_2)$, among them $I(\cdot)$ is indicative function.

1.2. MI-Based Sure Independence Screening Procedure

Let Y be a response vector and $X = (X_1^T,\cdots,\dots,X_p^T)^T$ be a vector of predictors, where each X_i is a $q_i \times 1$ vector for either grouped or categorical data for $i = 1,\cdots,p$. We defined the index sets of active and inactive predictors without specifying a statistical model as follows:

$$\mathrm{D} = \{k: P(Y|X_k)\ is\ nonconstant\ in\ X_k\ for\ some\ y \in \sigma(Y)\},$$
$$\mathrm{I} = \{k: P(Y|X_k)\ is\ a\ constant\ in\ X_k\ for\ any\ y \in \sigma(Y)\}. \qquad (6)$$

The main goal of variable selection is to find a set with fewer variables, which can contain the variable set D in the real model with probability 1. $\widehat{MI}(X,Y)$ is used as the variable screening indicator, by selecting the variable with a larger mutual information value between

the explanatory variable and the explained variable as the candidate set $\widehat{D}$, which is $\widehat{D} = \{k: \widehat{MI}(X_k, Y) \geq cn^{-\tau} \, for \, any \, 1 \leq k \leq p\}$, where c and τ are constants which are set in advance. There can be a simpler method in actual operation, namely $\widehat{D}^* = \{k: \widehat{MI}(X_k, Y) \, is \text{ the largest } d \text{ indicators}\}$.

Specifically, MI-SIS consists of two steps:

(i) calculate $\widehat{MI}(X_k, Y)$, which is an estimate of $MI(X_k, Y)$;

(ii) select the Xk that fall into $\widehat{D}^* = \{k: \widehat{MI}(X_k, Y) \, is \text{ the largest } 50 \text{ indicators}\}$.

1.3. Iterative MI-SIS

We used an iterative MI-SIS approach to enhance MI-SIS power which is similar to the work of Pan et al. [1]. Then, we used the Iterative Sure Independence Screening (ISIS) with with ten-fold cross-validation (CV) method and LASSO as the penalty function for intermediate penalized likelihood estimation to extract the features selected in the previous step.

References

1. Pan, W.; Wang, X.; Xiao, W.; Zhu, H. A Generic Sure Independence Screening Procedure. J Am Stat Assoc 2019, 114, 928-937, doi:10.1080/01621459.2018.1462709.
