# Supplementary figures and images for: TF–RBP–AS Triplet Analysis Reveals the Mechanisms of Aberrant Alternative Splicing Events in Kidney Cancer: Implications for Their Possible Clinical Use as Prognostic and Therapeutic Biomarkers
Source: Int J Mol Sci. 2021 Aug 16;22(16):8789. doi: 10.3390/ijms22168789 (PMC8395830; doi:10.3390/ijms22168789)

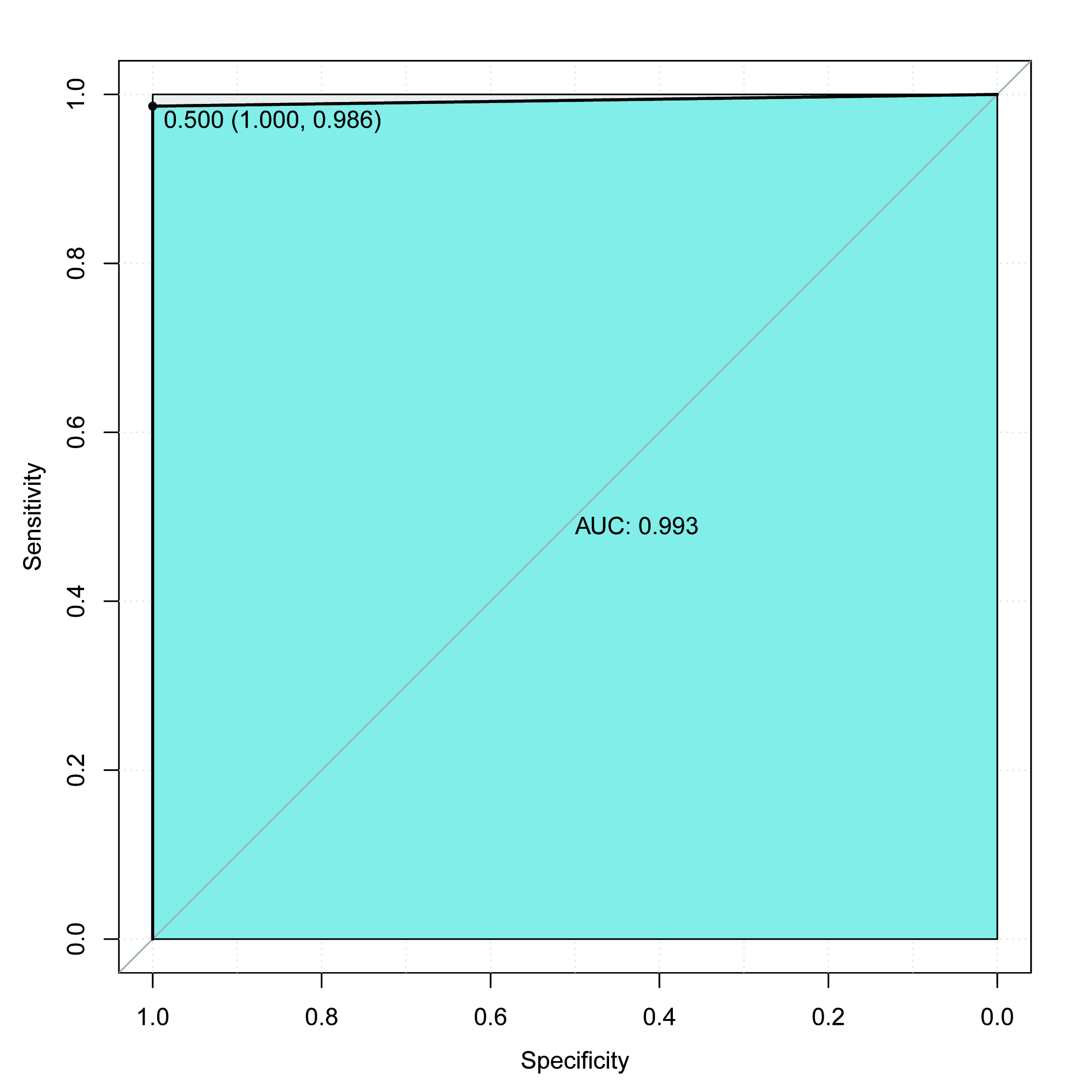

Supplement: Supplementary file 1 [file ijms-22-08789-s001.zip › Supplementary Figure S1ú║The receiver operating characteristic curve of 33 ASEs.tif]

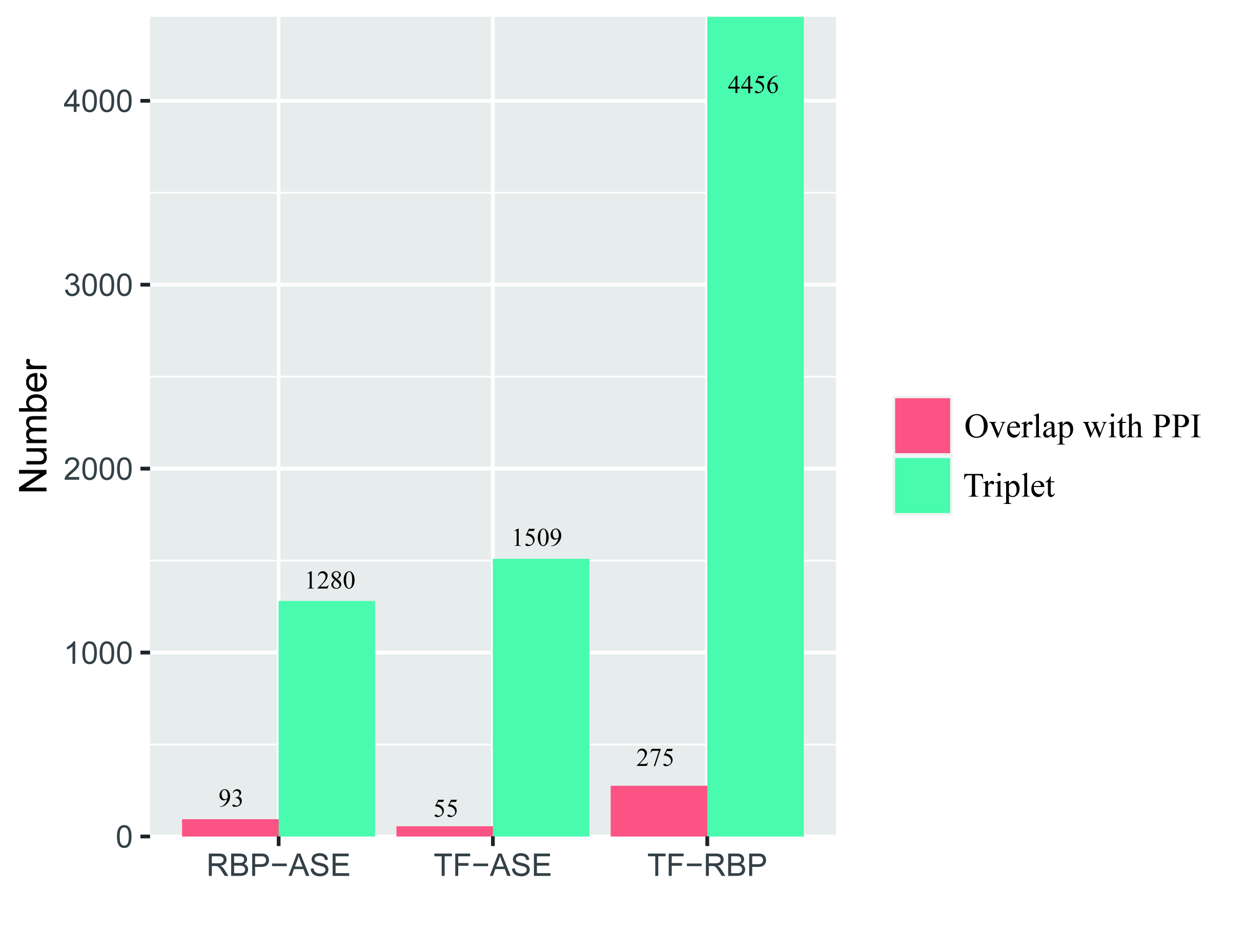

Supplement: Supplementary file 1 [file ijms-22-08789-s001.zip › Supplementary Figure S2ú║The gene pair relationship comparison of PPI and triplet.tif]

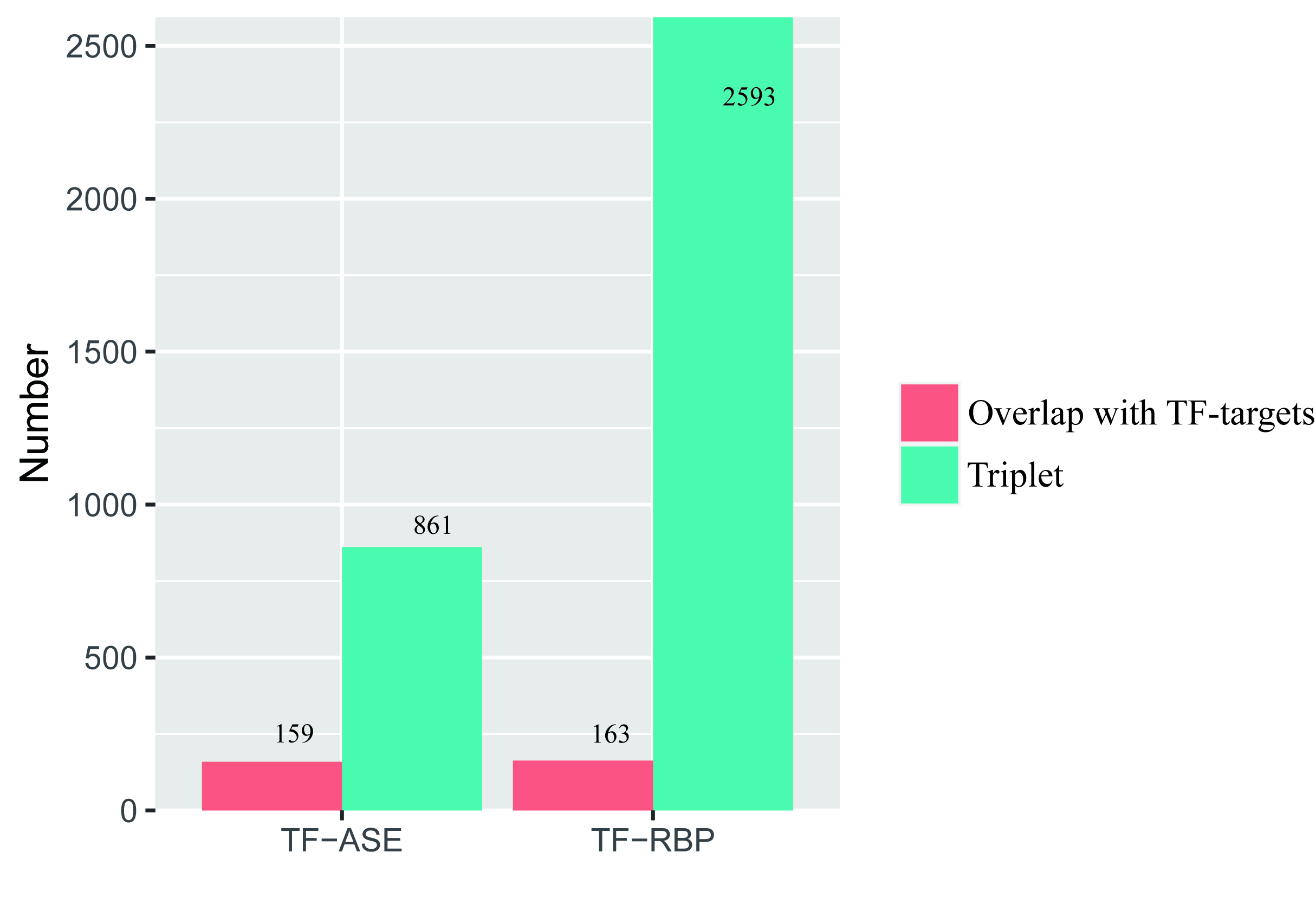

Supplement: Supplementary file 1 [file ijms-22-08789-s001.zip › Supplementary Figure S3ú║Alignment results of transcription factor target genes.tif]
